# Striving for scientific stringency: a re-analysis of a randomised controlled trial considering first-time mothers’ obstetric outcomes in relation to birth position

**DOI:** 10.1186/1471-2393-12-135

**Published:** 2012-11-22

**Authors:** Li Thies-Lagergren, Linda J Kvist, Kyllike Christensson, Ingegerd Hildingsson

**Affiliations:** 1 Department of Women’s and Children’s Health, Division of Reproductive Health, Karolinska Institutet, Stockholm SE-171 76, Sweden; 2 Department of Obstetrics and Gynaecology, floor 2, Helsingborg Hospital, Helsingborg SE- 25187, Sweden; 3 Department of Health Sciences, Lund University, Baravägen 3, Lund, Sweden; 4 Department of Health Sciences, Mid Sweden University, Holmgatan 10, Sundsvall SE- 851 70, Sweden

## Abstract

**Background:**

The aim of this study was to compare maternal labour and birth outcomes between women who gave birth on a birth seat or in any other position for vaginal birth and further, to study the relationship between synthetic oxytocin augmentation and maternal blood loss, in a stratified sample.

**Methods:**

A re-analysis of a randomized controlled trial in Sweden. An on-treatment analysis was used to study obstetrical outcomes for nulliparous women who gave birth on a birth seat (birth seat group) compared to birth in any other position for vaginal birth (control group). Data were collected between November 2006 and July 2009. The outcome measurements included perineal outcome, post partum blood loss, epidural analgesia, synthetic oxytocin augmentation and duration of labour.

**Results:**

The major findings of this paper were that women giving birth on the birth seat had shorter duration of labour and were significantly less likely to receive synthetic oxytocin for augmentation in the second stage of labour. Significantly more women had an increased blood loss when giving birth on the birth seat, but had no difference in perineal outcomes. Blood loss was increased regardless of birth position if women had been exposed to synthetic oxytocin augmentation during the first stage of labour.

**Conclusions:**

The results of this analysis imply that women with a straightforward birth process may well benefit from giving birth on a birth seat without risk for any adverse obstetrical outcomes. However it is important to bear in mind that, women who received synthetic oxytocin during the first stage of labour may have an increased risk for greater blood loss when giving birth on a birth seat. Finally it is of vital importance to scrutinize the influence of synthetic oxytocin administered during the first stage of labour on blood loss postpartum, since excessive blood loss is a well-documented cause of maternal mortality worldwide and may cause severe maternal morbidity in high-income countries.

**Trial registration:**

Unique Protocol ID: NCT01182038 ( http://register.clinicaltrials.gov)

## Background

Interventions in the childbirth process can, in both high- and low-income countries, save the lives of mothers and infants and in some cases reduce the occurrence of serious sequelae [1,2]. However, the general premise of current obstetric practice is that pregnancy and childbirth are medical processes; justifying intense risk management [3,4]. Medical–technical interventions are introduced into the birth process in what often becomes a “cascade of interventions”. Examples of such interventions are epidural analgesia, synthetic oxytocin for augmentation of labour, continuous electronic fetal monitoring and horizontal position in the second stage of birth [5,6]. It is well known that the majority of women in high-income countries, as well as in some low-income countries give birth in a horizontal position, despite repeated evidence for the numerous beneficial outcomes of upright birth positions [7-9].

It is more than 20 years since Waldenström and Gottvall [10] published results from a randomised controlled trial (RCT) of birth on a birth seat in a Swedish study that comprised 294 birthing women (compliance rate 49.3%). They reported from their study, which was analysed according to intention-to-treat (ITT), that participants in the experimental group had a significantly larger blood loss, a higher rate of maternal satisfaction and midwives attending birth seat births were less content with their working posture. A similar compliance rate (49.5%) was shown in a more recent Swedish RCT which reported obstetric outcomes in 1020 nulliparous women who were randomized to birth on a birth seat (experimental group) or to birth in any other position (control group) [9]. Analysis was by ITT. The main finding of the RCT was that there was no significant reduction in instrumental vaginal births in women allocated to the experimental group. Additionally, there was a significant increase in the experimental group for blood loss between 500 ml and 999 ml but no increase in bleeding above 1000 ml and no increase in perineal lacerations or perineal oedema [9]. A secondary analysis, also carried out according to the ITT principal, was performed to assess whether birth on the birth seat affected the use of synthetic oxytocin for augmentation of the second stage of labour, duration of labour and infant outcomes. The main findings of that analysis were that women allocated to the birth seat had a significantly shorter second stage of labour despite similar numbers of women who received synthetic oxytocin augmentation in both groups and that neonatal outcomes did not differ between the groups [11].

The randomized controlled trial is considered to be the most reliable method for empirical research and has in recent decades played an important role in producing evidence for midwifery and obstetric practice [12-14]. Johnson emphasizes, in “*Childbirth and Authoritative Knowledge”* edited by Davis-Floyd and Sarget [15], that the RCT is an important ally for supporting and further developing low-intervention, woman-centred midwifery care. According to some authors the most appropriate and recognized way to analyse results collected in an RCT is to perform an analysis according to the ITT principal [13,16]. When randomization is carried out meticulously the ITT analysis maintains the advantages of baseline comparability of the groups as well as balancing known and unknown confounders [17,18]. Nevertheless, analysis by the ITT principle in a sample with considerable non-compliance cannot demonstrate how an intervention affects the individual who complies with the allocation, since assessment will be diluted by the data from participants who do not receive the intervention to which they were randomly allocated [18]. Analysis according to the received treatment or “on-treatment” (OT) analysis is carried out without regard to the initial randomization and may be considered as a complement to ITT analysis when non-compliance is substantial, however with an increased risk for selection bias [18]. There was considerable non-compliance (49.5%) to randomization in the RCT by Thies-Lagergren et al. [9,11] and it is important to attempt to understand if the results, which showed a shortened second stage of labour and increased blood loss were associated with the use of the birth seat per se. Therefore, the present article is in response to colleagues’, researchers’ and reviewers’ questions about how the results of the maternal outcomes in the two ITT analyses [9,11] might be, if analysis was carried out according to how women actually gave birth (OT).

The aim of this re-analysis was to compare maternal labour and birth outcomes between women who gave birth on a birth seat or in any other position for vaginal birth and further, to study the relationship between synthetic oxytocin augmentation and maternal blood loss, in a stratified sample.

## Methods

The present article is based on data collected in an RCT by Thies-Lagergren et al. [9,11] and is a re-analysis of the following outcomes; synthetic oxytocin for augmentation of labour, perineal outcomes (episiotomies/lacerations/oedema), postpartum blood loss, duration of labour. An additional analysis regarding use of epidural analgesia was carried out.

### Design and trial size

This is an OT analysis of an RCT that was originally powered to detect differences in instrumental vaginal births, based on an arbitrary reduction of instrumental births from 15% to 9% (α = 0.05, ß = 0.2). Of the 1002 births analysed, fifty-two births were excluded due to emergency caesarean section, leaving 950 for analysis according to the OT principal. Analyses include 253 nulliparous women, referred to as the birth seat group, who gave birth on the birth seat compared to 697 nulliparous women, here referred to as the control group, who gave vaginal birth in any other position.

### Inclusion criteria

The participants spoke Swedish sufficiently well to receive information and give informed consent or refusal for participation in the trial. Inclusion criterion were a healthy woman with an uncomplicated pregnancy exclusive of any medical diagnosis, a singleton foetus in cephalic presentation, a Body Mass Index (BMI) less than 30 and a spontaneous onset of labour occurring between gestational weeks 37 + 0 and 41 + 6. Women diagnosed with gestational diabetes not requiring medical treatment were included. Also included were women with a history of caesarean section who now planned a vaginal birth (VBAC) and women induced because of spontaneous rupture of membranes without spontaneous contractions for longer than twenty-four hours.

### Recruitment of study participants

Midwives working in antenatal clinics invited women to join the study and provided oral and written information. Eligible women who accepted participation gave written consent and this was documented in the women’s case notes. The women were at liberty to withdraw their consent throughout the whole trial. The included women gave birth at two hospitals in Sweden, which were chosen for convenience. The assisting midwife, who checked that the inclusion criteria were met, confirmed eligibility for participation in the trial on admission to the delivery ward.

### Data collection

Data collection sheets contained the woman’s date of birth, personal identification number and randomization number. In case of non-compliance to randomization, the midwives were asked to record any reason for this on the data collection sheet. All other outcome measurements were available from the electronic case notes.

### Outcome measurements

The principal outcome measurements were post-partum blood loss and perineal outcome (episiotomies/lacerations/oedema).

Secondary outcomes were epidural analgesia, synthetic oxytocin for augmentation of first and second stage of labour and overall duration of labour. Infant outcomes are not reported in this paper since results of the previous analyses showed that very few infants (3.0 %) were transferred to the NICU.

### Statistical analyses

The data were analyzed using PASW (Predictive Analytics Software) version 20.0. Due to diversion from the original randomisation, a statistical comparison between the groups for demographic variables was carried out. We calculated odds ratios (OR) with a 95% confidence interval, for epidural analgesia, augmentation of labour, duration of labour, blood loss and perineal outcome between women who gave birth on the birth seat versus those who did not. Step-wise logistic regression analysis was used to examine the net effects of birth position and synthetic oxytocin for augmentation on blood loss greater than 500 ml. In the analysis the odds ratios were adjusted for maternal age, BMI, smoking, cervix status at admission, for epidural analgesia, fetal head circumference, fetal weight, gestational age, oxytocin augmentation and duration of first and second stage of labour.

### Ethical considerations

All participants included in the analysis were given standard midwifery care during birth. There is no evidence suggesting that giving birth on a birth seat should involve increased pain or medical risks for the birthing mother or her infant. There is an increased risk for blood loss between 500–999 ml, however, this has been shown to be without clinical relevance for participants [11]. The study has been approved by the committee for research ethics at Lund University [Dnr 214/2005 and Dnr 2009/739].

## Results

The group of 253 women in this analysis who gave birth on the birth seat comprises 246 (49.2%) of the 500 who were originally allocated to the experimental group and 7 (0.1%) from the control group who gave birth on the birth seat. All women who did not give birth on the birth seat, irrespective of allocation, are included in the control group. None in the birth seat group had an instrumental vaginal birth.

Table 1 shows demographic variables for the participants. There were significantly fewer women in the birth seat group who reported smoking.

**Table 1:** Demographic variables birth seat births compared to all other positions excl. ceaserean section

	**Birth seat group**	**Control group**	**RR/mean difference**	
	**n= 253**	**n= 697**	**(95% CI)**	**P-value**
	**n (%)**	**n (%)**		
**Age groups**				
< 25 years	**44 (17.4)**	**121 (17.3)**	**1.00 (0.73 - 1.37)**	**1.00**
25-35 years	**192 (75.1)**	**495 (71.1)**	**1.06 (0.98 - 1.16)**	**0.14**
> 35 years	**19 (7.5)**	**81 (11.6)**	**0.64 (0.40 - 1.04)**	**0.07**
**Body Mass Index (SD)**	**22.7 (±5.1)**	**22.7 (±5.1)**	**0.37 (0.80 - 6.65)**	**0.85**
**Smoking**	**22 (8.0)**	**95 (13.6)**	**0.63 (0.41 - 0.99)**	**0.04**
**Previous caesarean section**	**2 (0.7)**	**7 (1.0)**	**0.78 (0.16 - 3.76)**	**1.00**
**Gestational Age**				
< 37 + 6 weeks	**10 (3.9)**	**29 (4.1)**	**0.95 (0.47 - 1.91)**	**1.00**
38 + 0 - 40 + 6 weeks	**202 (79.8)**	**521 (74.7)**	**1.06 (0.99 - 1.15)**	**0.12**
> 41 + 0 weeks	**41 (16.2)**	**147 (21.0)**	**0.76 (0.56 - 1.08)**	**0.09**

Table 2 shows comparisons of labour outcomes between birth seat births and all other vaginal births. Statistically significantly differences were shown between the two groups; the birth seat group had shorter first (*p* = <0.01) and second (*p* = <0.01) stages of labour than the control group. These findings remain significant after adjustments. There was no difference between the groups for duration of the third stage of labour.

**Table 2:** Labour outcomes of birth seat births compared to all other positions (ceacarean section excluded)

	**Birth seat group**	**Control group**	**Crude**		**Adjusted**	
	**n= 253**	**n= 697**	**Odds Ratio**	**P-value**	**Odds Ratio**	**P-value**
	**n (%)**	**n (%)**	**(95% CI)**		**(95% CI)**	
**Duration of labour**						
First stage of labour in minutes*	**397 (± 191)**	**465 (± 240)**	**0.99 (0.99 - 0.99)**	**<0.01**	**0.99 (0.99 - 1.00)**	**<0.01**
Second stage of labour in minutes**	**32 (± 18)**	**44 (± 27)**	**0.97 (0.70 - 0.98)**	**<0.01**	**0.97 (0.96 - 0.98)**	**<0.01**
Third stage of labour in minutes***	**14 (± 17)**	**13 (± 14)**	**1.00 (0.99 - 1.03)**	**0.34**	**1.00 (0.99 - 1.01)**	**0.22**
**Augmentation of labour**						
No augmentation	**127 (50.2)**	**202(28.9)**	**1.0 Ref.**		**1.0 Ref.**	
Augmentation initiated during first stage of labour****	**90 (35.6)**	**355 (50.9)**	**0.40 (0.29 - 0.56)**	**<0.01**	**0.74 (0.49 - 1.13)**	**0.17**
Augmentation initiated during second stage of labour*****	**36 (14.2)**	**140 (20.1)**	**0.66 (0.44 - 0.98)**	**0.04**	**0.49 (0.29 - 0.86)**	**<0.01**
Epidural analgesia******	**94 (37.0)**	**314 (45.0)**	**0.72 (0.54 - 0.97)**	**0.03**	**1.14 (0.75 - 1.73)**	**0.54**
Blood loss*******						
< 499 ml	**86 (34.0)**	**355 (51.1)**	**1.0 Ref.**		**1.0 Ref.**	
500-999 ml	**128 (50.6)**	**246 (35.1)**	**1.43 (1.22 - 1.68)**	**<0.01**	**2.20 (1.48 - 3.26)**	**<0.01**
>1000 ml	**39 (15.4)**	**96 (13.8)**	**1.67 (1.08 - 2.60)**	**0.02**	**2. 00 (1.19 - 3.37)**	**<0.01**
Mean blood loss in ml. (SD)*******	**709 (± 462)**	**592 (± 448)**	**117**	**<0.01**	**1.00 (1.00 - 1.01)**	**<0.01**
Episiotomies*******	**5 (2.0)**	**90 (13.7 )**	**0.15 (0.06 - 0.37)**	**<0.01**	**0.19 (0.07 - 0.54)**	**<0.01**
Perineal lacerations*******						
No laceration	**10 (4.0)**	**40 (6.1)**	**1.0 Ref.**		**1.0 Ref.**	
1 degree	**184 (73.8)**	**458 (69.8)**	**1.06 (0.97 - 1.15)**	**0.25**	**1.63 (0.70 - 3.80)**	**0.26**
2.degree	**42 (16.8)**	**118 (17.9)**	**1.93 (0.68 - 1.30)**	**0.77**	**1.53 (0.60 - 3.89)**	**0.37**
3.degree	**13 (5.2)**	**40 (6.1)**	**0.85 (0.47 - 1.57)**	**0.75**	**1.76 (0.60 - 5.29)**	**0.31**
Oedema*******						
1 (vas 0-3)	**184 (85.2)**	**419 (83.4)**	**1.0 Ref.**		**1.0 Ref.**	
2 (vas 4-7)	**30 (13.8)**	**70 (13.9)**	**0.99 (0.67 - 1.48)**	**1.00**	**1.79 (0.35 - 8.77)**	**0.51**
3 (vas 8-10)	**2 (0.9)**	**13 (2.6)**	**0.35 (0.08 - 1.57)**	**0.25**	**2.54 (0.48 - 13.64)**	**0.28**

* Adjusted for maternal age, BMI, smoking, cx status at admission, epidural, fetal head circumference, fetal weight, gestational age.

** Adjusted for maternal age, BMI, smoking, cx status at admission, epidural, fetal head circumference, fetal weight, gestational age, duration of first stage of labour and augmentation during first stage.

***Adjusted for maternal age, BMI, smoking, cx status at admission, epidural, fetal head circumference, fetal weight, gestational age, duration of first and second stages, augmentation during first and second stages.

**** Adjusted for maternal age, BMI, smoking, cx status at admission, epidural, fetal head circumference, fetal weight, gestational age and duration of first stage of labour.

*****Adjusted for maternal age, BMI, smoking, cx status at admission, epidural, fetal head circumference, fetal weight, gestational age, oxytocin augmentation in first stage of labour and duration of first and second stages of labour.

******Adjusted for maternal age, BMI, smoking, cx status at admission, fetal head circumference, fetal weight, gestational age, oxytocin augmentation in/and duration of first and second stages of labour.

******* Adjusted for maternal age, BMI, smoking, cx status at admission, epidural, fetal head circumference, fetal weight, gestational age, oxytocin augmentation in/and duration of first and second stages of labour.

A comparison between the groups for labour augmentation initiated during the first and second stages of labour showed that less women in the birth seat group were given synthetic oxytocin for labour augmentation during both the first stage (*p* = <0.01) and the second stage (*p* = 0.05) of labour. However after adjustment only augmentation in the second stage of labour remained statistically significant.

Epidural analgesia was administered to 37% of the women in the birth seat group compared to 45% in the control group, which represented a statistically significant difference, but when adjusted for potential confounders the difference disappeared.

Statistically significantly more women who gave birth on the birth seat had a measured blood loss of 500 ml or more. The odds for increased blood loss were higher even after adjustment.

Among the total number of participating women, 14.7 % had a documented blood loss more than 1000 ml. In order to more fully understand the relationship between blood loss and augmentation, a stratified analysis was performed between the birth seat group and the control group and results are shown in Table 3. This analysis showed that there was no difference for blood loss from 500 to 999 ml between those with or without augmentation during the first stage of labour, in the birth seat group. However, those in the birth seat group who received synthetic oxytocin significantly more often bled over 1000 ml (*p* = 0.04).

**Table 3:** Stratified analysis of blood loss in relation to augmentation with synthetic oxytocin

		**Birth seat group**			**Control group**	
	**No oxytocin initiated**	**Oxytocin initiated**	**OR (95% CI)**	**No oxytocin initiated**	**Oxytocin initiated**	**OR (95% CI)**
	**during first stage**	**during first stage**	**for oxytocin during**	**during first stage**	**during first stage**	**for oxytocin during**
**Blood loss**	**n (%)**	**n (%)**	**first stage**	**n (%)**	**n (%)**	**first stage**
<499 ml	63 (38cif.6)	23 (25.5)	1.0 Ref.	193 (56.4)	162 (45.5)	1.0 Ref.
500-999 ml	79 (48.6)	49 (54.5)	1.7 (0.9-3.1)	113 (33.0)	133 (37.0)	1.4 (1.0 -1.9)**
>1000 ml	21 (12.8)	18 (20.0)	2.3 (1.1-5.1)*	36 (10.6)	60 (17.5)	2.0 (1.2-3.1)**
	**No oxytocin initiated**	**Oxytocin given**	**OR (95% CI)**	**No oxytocin given**	**Oxytocin given**	**OR (95% CI)**
	**during second stage**	**during second stage**	**for oxytocin during**	**during second stage**	**during second stage**	**for oxytocin during**
**Blood loss**	**n (%)**	**n (%)**	**second stage**	**n (%)**	**n (%)**	**second stage**
<499 ml	51 (40.0)	12 (33.4)	1.0 Ref.	120 (59.0)	73 (52.1)	1.0 Ref.
500-999	59 (46.5)	20 (55.6)	1.5 (0.6-3.2)	62 (31.0)	51 (36.4)	1.4 (0.8-2.2)
>1000	17 (13.5)	4 (11.0)	1.0 (0.3-3.5)	20 (10.0)	16 (11.5)	1.3 (0.6-2.7)

* = p <0.05, ** = p <0.01.

In the control group there were significantly more women who had a blood loss between 500 – 999 ml (*p* = 0.04) and significantly more who had a blood loss over 1000 ml (*p* = 0.003) when they had received synthetic oxytocin during first stage of labour.

Perineal outcomes are also shown in Table 2. Among the women who gave birth on the birth seat five (2%) had an episiotomy performed compared to 90 (13.7%) in the control group, which was statistically significant (*p* = <0.01). There were no statistically significant differences for any degree of perineal lacerations. Oedema was measured on a visual analogue scale (VAS) between 24–36 hours after birth and no difference was demonstrated between the groups.

## Discussion

The major findings in this paper were that women giving birth on the birth seat had shorter duration of labour and were significantly less likely to receive synthetic oxytocin for augmentation in the second stage of labour. There were no differences in perineal outcomes between the groups. Significantly more women had an increased blood loss when giving birth on the birth seat. Blood loss was increased regardless of birth position if women had been exposed to synthetic oxytocin augmentation during the first stage of labour.

These findings altogether reveal the complexity of interventions used in contemporary obstetric practice; one might ask: what came first, the chicken or the egg? It may be speculated that women who gave birth on the birth seat had a more straightforward labour, were less tired and experienced less pain, making them less exposed to interventions. It is known that upright birth positions improve contractions, make pain easier to handle and enhance shorter duration of labour, and should therefore be used as an intervention to facilitate a straightforward birth [19-23]. Experiences of physiological birth may enhance midwives trust in the birth process and lessen the tendency for intervention. On the other hand, women whose labours are not straightforward may request a more medical approach from the midwife, wishing for the introduction of interventions to prevent prolonged labours and resulting dissatisfaction with childbirth.

According to the ITT analysis the second stage of labour was significantly shorter for women who gave birth on the birth seat and when the OT analysis was applied, it was disclosed that the overall duration of labour for those women was shorter and duration of the second stage of labour was even shorter (ITT = 6 min vs. OT = 12 min).

Analysis by ITT found no statistically significant difference between the groups for blood loss above 1000 ml but according to the OT analysis, significantly more women who gave birth on the birth seat had a blood loss of ≥1000 ml. In accordance with earlier research, the present analysis suggests that upright position facilitated by the birth seat may cause a greater blood loss, however the increased blood loss was of little clinical relevance for the women, as reported earlier [8-10]. The greater blood loss may be due to venous obstruction or be caused by increased hydrostatic pressure both on the arterial and venous side, which could contribute to more bleeding from the uterus and placental site [10,24]. Another possible explanation for blood loss above 500 ml may be found in the use of synthetic oxytocin for augmentation. According to the ITT analysis no differences were found between the groups regarding synthetic oxytocin for labour augmentation during either the first or second stage of labour. However, according to the OT analysis statistically significantly less women in the birth seat group received augmentation during second stage of labour. The stratified analysis showed a statistically significant association between blood loss and augmentation of labour with synthetic oxytocin during the first stage of labour regardless of group affiliation. Prolonged labour often results in augmentation of labour and an increased risk for post partum blood loss [25], which may even be the case in this study. However, a population-based study stated that synthetic oxytocin during labour appears to be an independent risk factor for increased blood loss, regardless of labour duration [26]. Synthetic oxytocin is a commonly used drug in contemporary obstetrics; it is of vital importance to further investigate the influence of its administration during the first stage of labour on postpartum blood loss.

Regarding perineal lacerations and perineal oedema this analysis is consistent with the ITT analysis. There were no increased incidences in first, second or third degree perineal lacerations for women giving birth on a birth seat, which is in contrast to earlier findings from a systematic Cochrane-review [9,22]. Moreover, it was shown in the present analysis that significantly fewer women who gave birth on the birth seat had an episiotomy performed. This is an important finding that may be linked to less interventions and the reduced length of the second stage of labour in these women. Gupta et al., [22] reported likewise that upright position in the second stage of labour for women without epidural analgesia resulted in a considerable reduction in episiotomy.

In hospitals, trust in medical guidelines rather than the physiological process of birth is common [27]. Some researchers have discussed how midwives attitudes to labour and birth might have an impact on labour progress [28]. The present analysis has not investigated midwives attitudes, nevertheless is it important to note that our analysis removes non-compliers, which might mean that the women who gave birth on the birth seat were attended by a midwife who was generally more positive to the idea of upright birthing positions.

### Methodological considerations

It is a great challenge to conduct and achieve high compliance in RCTs carried out in an intrapartum care setting [29]. The primary reason for the present analysis was the substantial non-compliance, which occurred in the RCT, making the ITT approach challenging. It has been suggested that full ITT analysis is only meaningful when complete outcome data are available for all subjects included in an RCT [16,18]. In our case, data were complete but the substantial non-compliance prompted us to consider an alternative analysis. An OT analysis answers questions about true effect by analysing the received intervention rather than the allocated intervention [18]. However, it has also been suggested that although OT analysis may provide clinically relevant information and valuable clarifications in the assessment of interventions, ITT remains the most reliable way to interpret analyses of RCTs [19]. The present OT analysis of the data originating from the RCT has inherent limitations because of the loss of the benefits of randomization and a risk for selection bias. However, this issue was in part addressed by carrying out a statistical comparison of demographic data between the groups.

It is important to bear in mind that the women who participated in the trial probably had a positive attitude towards the birth seat when they initially agreed to join the study. The results from the OT analysis might also have pin-pointed those women who were motivated to give birth without interventions and who wanted to and actually gave birth on the birth seat. Further research to understand the negotiation process between the midwife and the birthing woman will be of great interest.

Our results indicate that it may be relevant to combine the two methods of analysis when compliance is reduced. In a letter to the editor of the journal “Infection Control and Hospital Epidemiology”, Herigon & Newland [30] suggested that both a strict ITT and an OT analysis should be used to inform the overall conclusions of any given randomized trial. Both analyses provide estimates of the true effect, which likely lies somewhere between the two estimates, while offering different trade-offs.

## Conclusion

We have illustrated that non-compliance in intra-partum studies remains a problem and that choice of method of analysis has a considerable impact on results obtained. This fact leads us to reflect on cause and effect: the chicken or the egg? Results of this analysis imply that women with a straightforward birth process may well benefit from giving birth on a birth seat without risk for any adverse obstetrical outcomes. However it is important to bear in mind that women who receive synthetic oxytocin during the first stage of labour, may have an increased risk for blood loss ≥ 1000 ml, regardless of birth position. Finally it is of vital importance to scrutinize the influence of synthetic oxytocin administered during the first stage of labour on blood loss postpartum, since excessive blood loss is a well-documented cause of maternal mortality worldwide and may cause maternal morbidity even in high-income countries.

## Competing interests

The authors declare that they have no competing interests.

## Authors’ contributions

LTL conducted the trial and drafted the manuscript. LJK made substantial contribution in planning the trial, drafting the manuscript and analysing data. KC revised the manuscript critically and gave final approval for the version to be published. IH made substantial contribution in analysis and interpretation of data. All authors read and approved the final manuscript.

## Pre-publication history

The pre-publication history for this paper can be accessed here:

http://www.biomedcentral.com/1471-2393/12/135/prepub
